# Potential Anti-Allergy and Immunomodulatory Properties of *Lactococcus lactis* LB 1022 Observed In Vitro and in an Atopic Dermatitis Mouse Model

**DOI:** 10.4014/jmb.2301.01019

**Published:** 2023-03-08

**Authors:** Jihye Baek, Jong-Hwa Kim, Wonyong Kim

**Affiliations:** 1 Department of Microbiology, College of Medicine, Chung-Ang University, Seoul 06974, Republic of Korea; 2 LuxBiome Co., Ltd., Seoul 06974, Republic of Korea

**Keywords:** *Lactococcus lactis*, atopic dermatitis, allergic inflammation, Th2, ovalbumin

## Abstract

*Lactococcus lactis* is a lactic acid bacterium and used in the dairy food industry. The ameliorating effects of *Lactobacillus* species on atopic dermatitis (AD) have been extensively studied, but the specific effect of *L. lactis* strains has not yet been investigated. In this study, the efficacy of *L. lactis* LB 1022, isolated from natural cheese, was evaluated using RAW 264.7, HMC-1 and HaCaT cell lines and an ovalbumin-sensitized AD mouse model. *L. lactis* LB 1022 exhibited nitric oxide suppression and anti-allergy and anti-inflammatory activity in vitro. Oral administration of *L. lactis* LB 1022 to AD mice significantly reduced the levels of IgE, mast cells, and eosinophils, and a range of T cell-mediated T helper Th1, Th2, and Th17-type cytokines under interleukin (IL)-10, transforming growth factor-β (TGF-β), thymus and activation-regulated chemokine (TARC), and thymic stromal lymphopoietin (TSLP). In addition, *L. lactis* LB 1022 treatment increased the concentration of short-chain fatty acids. Overall, *L. lactis* LB 1022 significantly modulated AD-like symptoms by altering metabolites and the immune response, illustrating its potential as candidate for use in functional food supplements to alleviate AD.

## Introduction

Atopic dermatitis (AD) is a common chronic relapsing skin disorder, affecting at least 230 million people globally [1]. Though various genetic and environmental factors and the Western diet have been associated with AD, its pathogenesis remains difficult to fully understand [2]. Recent studies have reported that the pathogenesis of AD is related to gut dysbiosis with inflammation and imbalanced immune responses [3]. In this context, lactic acid bacteria, which are commonly used as probiotics, have been shown to improve gut health by regulating gut and immune homeostasis and alleviating allergic disease in the host. In particular, *Lactobacillus* spp. and *Bifidobacterium* have been proposed as complementary therapeutic agents for AD [4-6].

*Lactococcus lactis*, which has been classified by the Food and Drug Administration, is widely used as a starter culture to produce dairy products including cheese, fermented milk products, and sour cream [7]. *L. lactis* subsp. *lactis* is also found in dairy products and fermentation processing [8]. It has been found that oral administration of *L. lactis* strain C59 decreases IgE levels in inflammation in ovalbumin (OVA)-induced mice models, while a recent study reported that *L. lactis* strain NZ9000 prevents airway OVA-induced asthmatic mice [9, 10]. However, other studies have demonstrated the effects of *L. lactis* on AD symptoms.

This study thus investigated the anti-allergy and anti-inflammatory properties of *L. lactis* LB 1022 in OVA-induced mice and whether these properties are linked to alters in the function of short-chain fatty acid (SCFA) metabolites and to systemic immunomodulatory response, with the ultimate aim to identify a new probiotic candidate for the treatment of AD.

## Materials and Methods

### Cultures of *Lactococcus lactis* LB 1022

To isolate *L. lactis* subsp. *lactis*, a cheese sample was serially diluted to a concentration of 10^-7^, added to trypticase soy agar (BD BBL, USA), and left at 30°C for 48 h for use as a selective medium [11]. Following the *L. lactis* LB 1022 isolation process, colonies were selected and identified using 16S rRNA gene sequencing [12]. The isolate was placed in trypticase soy broth (BD BBL) containing 50 % (v/v) glycerol for preparing stock and stored at –80°C.

### Cell Cultures

Human mast cell line (HMC-1), human keratinocyte cell line (HaCaT), and murine macrophages cell line (RAW 267.4) were purchased from the Korean Cell Line Bank (Korea). HMC-1 were cultivated in Iscovés Modified Dulbeccós Medium (Lonza, Switzerland) added with 10% MSC-qualified fetal bovine serum (FBS)(Gibco BRL, USA), HaCaT in Dulbecco’s Modified Eagle’s Medium (DMEM; Lonza) containing 10% FBS, and RAW 264.7 cells in DMEM supplemented with 10% FBS. All three cultures were kept at 37°C in 5% CO_2_ incubator.

### Anti-Inflammatory Activity

RAW 264.7 cells were used to investigate the effect of *L. lactis* LB 1022 on the immune response. RAW 264.7 cells were seeded at 5.0 × 10^5^ cells/well and pretreated with *L. lactis* LB 1022 (1 × 10^8^ CFU) for 1 h prior to stimulation with 0.1 μg/ml of lipopolysaccharide (LPS), followed by incubation for 24 h. The NO concentration was assessed using Griess reagent (Sigma-Aldrich, USA) and measured using a microplate reader (Infinite M200 Nano-quant; Männedorf, Switzerland) at a wavelength of OD_540_ [13]. The MTT cell growth assay reagent 3-(4,5-dimethylthiazol-2-yl)-2,5-diphenyltetrazolium bromide (Sigma–Aldrich) was measured the effect of *L. lactis* LB 1022 on the viability [14]. RAW 264.7 cells were seeded at 5.0 × 10^5^ cells/well for 24 h. *L. lactis* LB 1022 (1 × 10^8^ CFU) was replaced to fresh DMEM and incubated for 24 h. Then, 500 μl of 5 mg/ml MTT reagent was added, and incubation for 1 h. Then, 500 μl of dimethyl sulfoxide (Sigma-Aldrich) added, and the cells then measured at OD_590_.

### Thymus and Activation-Regulated Chemokine (TARC) and T helper 2 (Th2) Cytokine Assays for HaCaT Cells

HaCaT cells were seeded at 5.0 × 10^5^ cells/well and treated with *L. lactis* LB 1022 (1 × 10^8^ CFU) and stimulated with tumor necrosis factor (TNF)-a/ interferon (IFN)-g (both 20 ng/ml) for 24 h. The supernatant was centrifuged at 1,000 ×*g* for 5 min. The secretion levels of TARC and Th2-related cytokines (IL-4, IL-5, and IL-13) in the HaCaT cells were performed using an ELISA kit (BD Biosciences, USA).

### Measurement of β-Hexosaminidase and Histamine Release Assays

Inhibitory effects on release and activity from HMC-1 cells were performed as previously described [15]. Briefly, to the β-hexosaminidase inhibition assays, HMC-1 cells were seeded at 2 × 10^6^ cells/well and added Tyrode’s buffer (0.4 mM NaH_2_PO_4_, 1.8 mM CaCl_2_, 2.7 mM KCl, 5.6 mM glucose, 11.9 mM NaHCO_3_, and 137 mM NaCl; pH 7.2) and *L. lactis* LB 1022 (10^8^ CFU/ml) for 30 min. Samples of 20 μl were then incubated at 37°C for 1 h with 30 μl of 1 mM *p*-nitrophenyl-N-acetyl-β-D-glucosaminide suspended in a 0.1 M citrate buffer. Finally, the samples were terminated to stop the reaction. The absorbance was measured at OD_405_ using an Infinite M200 microplate reader (Nano-quant). Histamine assay was conducted using a histamine ELISA kit (Cayman Chemical Company, USA). The absorbance was measured at OD_405_ with an Infinite M200 microplate reader (Nano-quant) and compared with a standard curve to identify the histamine levels.

### Animal Studies

Female BALB/c mice (5 weeks old) were obtained from Central Laboratory Animal in Korea (Korea). The animals were maintained in hard-top cages (five mice per cage) under a 12-h light/dark cycle at a temperature of 22 ± 2°C and humidity of 50 ± 10%. The experiments were approved by the Animal Care Committee of Chung-Ang University (IACUC no. 2018-00022). The mice were distributed into three groups (*n* = 5 / group) as follows: control, OVA (Sigma-Aldrich; the AD-induced group), and *L. lactis* LB 1022 (AD + treatment with *L. lactis* LB 1022). To sensitive an AD immunological response and skin lesions, once a week, the hair on the dorsal skin of the mice was shaved. Six-week-old BALB/c mice were sensitized intraperitoneally with OVA (50 μg/ml) and alum (Sigma-Aldrich) at 1, 3, 5, and 7 weeks. The LB 1022 group received *L. lactis* LB 1022 (1 × 10^8^ CFU) orally using a gavage for 8 weeks.

### Dermatitis Scoring

The dermatitis score of AD on the dermal was scored twice a week as the sum of individual erythema, dryness, edema, and erosion/excoriation scores (0, none; 1, mild; 2, moderate; and 3, severe).

### Quantitation of Serum IgE and Cytokine Levels

The levels of total IgE. IL-4, IL-5, IL-12, IL-10, IL-13, thymic stromal lymphopoietin (TSLP), IFN-γ, TNF-α, in the serum were measured using ELISA kits (R&D Systems; Abcam, USA). The absorbance was measured at OD_450_ and compared with a standard curve to calculate the cytokine levels.

### Real-Time Quantitative PCR Analysis

Total RNA was extracted from skin material by a RNeasy Mini Kit (Qiagen, Germany) and the cDNA was synthesized using a PrimeScript 1st strand cDNA Synthesis Kit (Takara, Japan) following the manufacturer’s instructions and quantitatively analyzed with SYBR Green (Qiagen) using a QuantStudio 3 system. The 36B4 housekeeping gene was used as control. The primers used were presented in Table 1.

### Histological Analysis

The dorsal skin tissue was collected and fixed with 10% formaldehyde solution and embedded in paraffin. Paraffin blocks were cut to a thickness of 4–5 μm and stained with hematoxylin and eosin solution (H&E; Sigma-Aldrich) for inflammation and Toluidine Blue solution (Sigma-Aldrich) for mast cells under a DM 4000B microscope (Leica Microsystem, Wetzlar).

### Short-Chain Fatty Acid Analysis

To determine whether the SCFA levels are different between control, LB 1022, and OVA, stool samples were collected from each mouse at 8, 12, and 16 weeks and stored at −80°C. The samples were reconstituted in deionized water and passed through a membrane filter using Sep-Pak Vac cartridges (WAT054955; Waters, USA). Filtered samples were then analyzed using high-performance liquid chromatography (HPLC) by a refractive index detector (RefractoMAX520; ERC, Japan).

### Statistical Analysis

All data were expressed as the mean ± SEM. GraphPad Prism (v.8.0) was used to visualize and analyze the data using Tukey’s multiple comparison tests. In vitro data were performed using Student’s t-test. Statistical significance is denoted as ***p < 0.001, ***p < 0.0001.

## Results

### *L. lactis* LB 1022 Suppresses the Production of NO Induced by LPS in RAW264.7 Cells

**Fig. 1:**
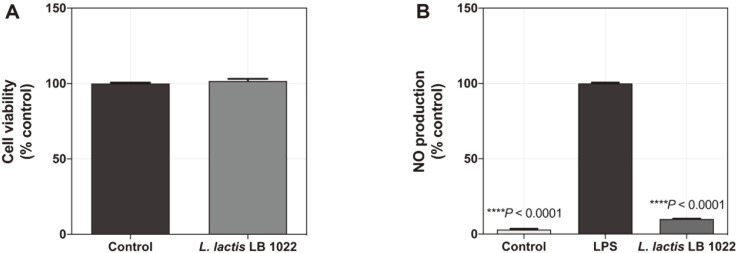
Effects of oral administration of *L. lactis* LB 1022 on the viability and NO production. (**A**) RAW264.7 cells were incubated with the indicated doses of *L. lactis* LB 1022 (1 × 10^8^ CFU) for 24 h, and cell viability was performed by MTT assay. (**B**) RAW 264.7 cells were pre-incubated with *L. lactis* LB 1022 for 4 h, and then treated with 1 μg/ml LPS for an additional 24 h. NO production was analyzed using the Griess reaction. Statistical differences were assessed between OVA and Control/*L. lactis* LB 1022 by one-way ANOVA by Turkey’s post hoc tests as the mean ± SEM.

To evaluate the cell viability effect of *L. lactis* LB 1022, RAW 264.7 cells were incubated with *L. lactis* LB 1022 at the determined concentrations for 24 h. *L. lactis* LB 1022 was not cytotoxic to RAW 264.7 cells tested at 1 × 10^8^ CFU with the MTT assay (Fig. 1). Next, to investigate the inflammatory effects of *L. lactis* LB 1022, the NO production by RAW264.7 cells due to LPS was assessed as an in vitro model. The results indicated that LPS significantly increased the NO production in RAW 264.7 cells (****p < 0.0001 vs. LPS), whereas *L. lactis* LB 1022 treatment significantly decreased (****p < 0.0001 vs. LPS; Fig. 1B).

### *L. lactis* LB 1022 Exhibits Anti-Allergy and Anti-Inflammatory Activity

**Fig. 2:**
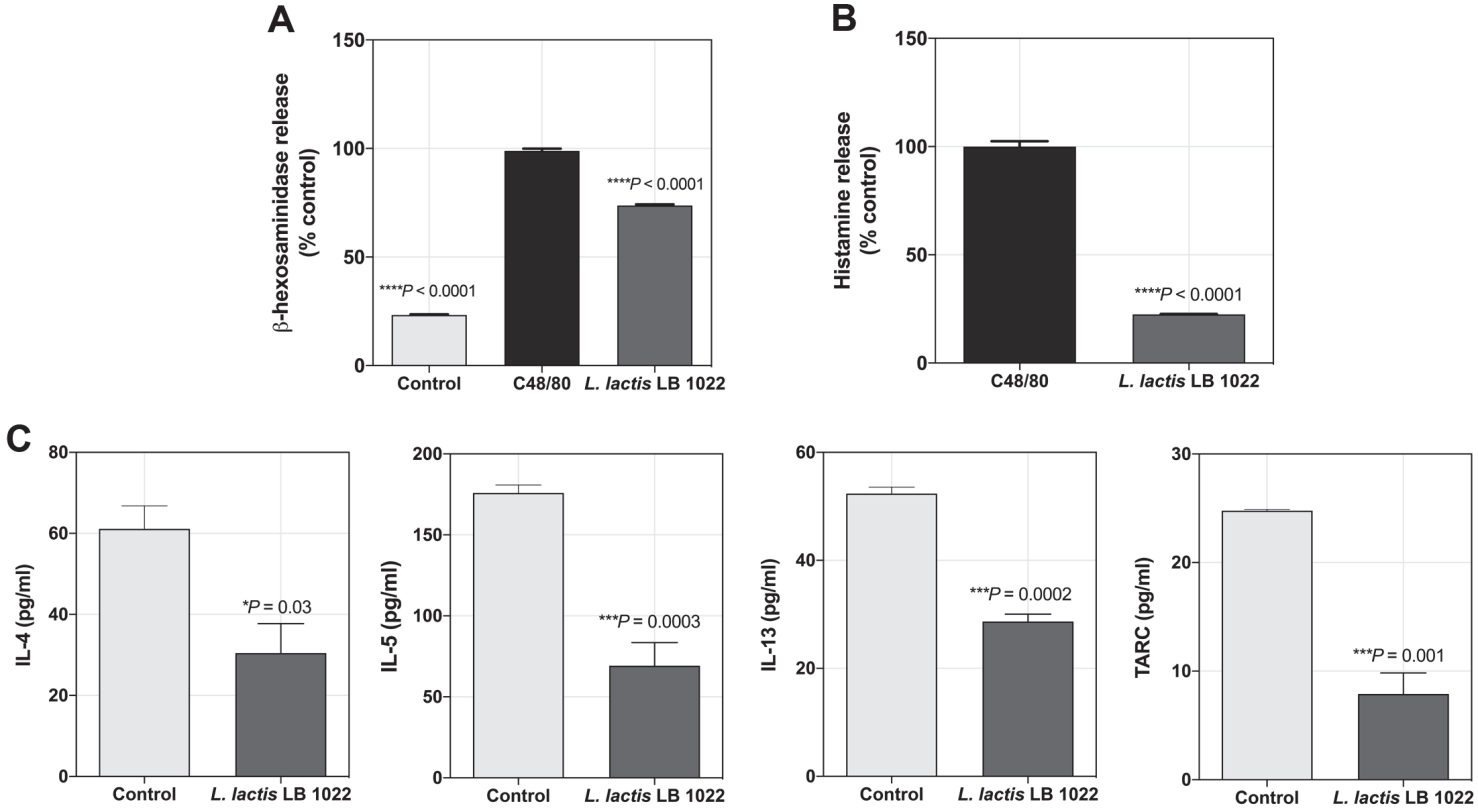
Inhibitory effects of *L. lactis* LB 1022 on β-hexosaminidase, histamine assays, and Th2-mediated inflammation. (**A**) β-hexosaminidase and (**B**) histamine release assays for HMC-1 cells. (**C**) Levels of chemokine (TARC) and Th2-related cytokines (IL-4 and IL-13) in TNF-α/IFN-γ-treated in HaCaT cells. Statistical differences between the control and *L. lactis* LB 1022 groups were assessed using unpaired 2-tailed t-tests or one-way ANOVA followed by Turkey’s post-hoc tests (mean ± SEM).

To examine whether *L. lactis* LB 1022 has anti-allergic activity, the production of β-hexosaminidase was monitored. Untreated and pre-treated with *L. lactis* LB 1022 for 30 min were challenged with activator C48/80 (Sigma-Aldrich). The stimulated HMC-1 cells treated with *L. lactis* LB 1022 had β-hexosaminidase levels that were 73.7 ± 0.5% higher than that of the control (Fig. 2A). To determine whether *L. lactis* LB 1022 had a regulatory effect on the degranulation of mast cells, the activity of histamine was tested. The results revealed that *L. lactis* LB 1022 significantly reduced the release of histamine (Fig. 2B). In addition, *L. lactis* LB 1022 suppressed cytokine levels of IL-4, IL-5, IL-13, and TARC in HaCaT cells (Fig. 2C), indicating that *L. lactis* LB 1022 inhibited TARC activation, leading to the downregulation of Th2 cytokines.

### *L. lactis* LB 1022 Treatment Alleviates Clinical AD Symptoms in OVA-Induced AD Mice

**Fig. 3:**
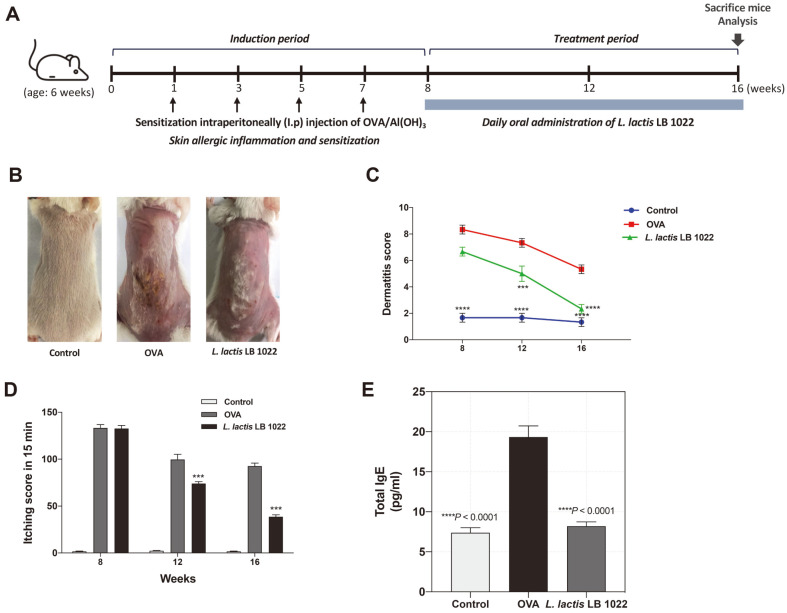
Changes in symptoms following oral administration of *L. lactis* LB 1022 in mice with OVA-induced atopic dermatitis (AD)-like symptoms. (**A**) Experimental design. The mice were sensitized with OVA for 8 weeks. After 8 weeks, the mice were fed LB 1022 for an additional 8 weeks. Dermatitis scores were measured at 4-week intervals. Statistical differences between the OVA and control/*L. lactis* LB 1022 groups were assessed using one-way ANOVA followed by Turkey’s post-hoc tests (mean ± SEM). *** *p* < 0.001; *** *p* < 0.0001.

The animal experimental process is illustrated in Fig. 3A. *L. lactis* LB 1022 mice group significantly reduced skin symptoms in OVA-induced AD mice model (Fig. 3B). The dermatitis scores in the *L. lactis* LB 1022 group were significantly decreased than OVA group (***p = 0.0008 at 12 weeks; ****p < 0.0001 at 16 weeks) (Fig. 3C). Itching behaviour was also monitored, and it was found that repeated topical application of *L. lactis* LB 1022 decreased the itchiness scores compared with OVA group after 12 weeks (***P = 0.0008), with a 2-fold decrease after 16 weeks (***p = 0.0002; Fig. 3D). Furthermore, total IgE was promoted in the OVA group compared with the control and *L. lactis* LB 1022 groups (Fig. 3E), which suggests that *L. lactis* LB 1022 leads to the inhibition of AD-like skin symptoms and AD pathology in OVA-induced AD mice.

### *L. lactis* LB 1022 Treatment Alleviates Atopic Dermatitis Symptoms by Decreasing Mast Cell and Eosinophils Infiltration and Regulatory Epithelium-Derived Innate Cytokines in the Skin

**Fig. 4:**
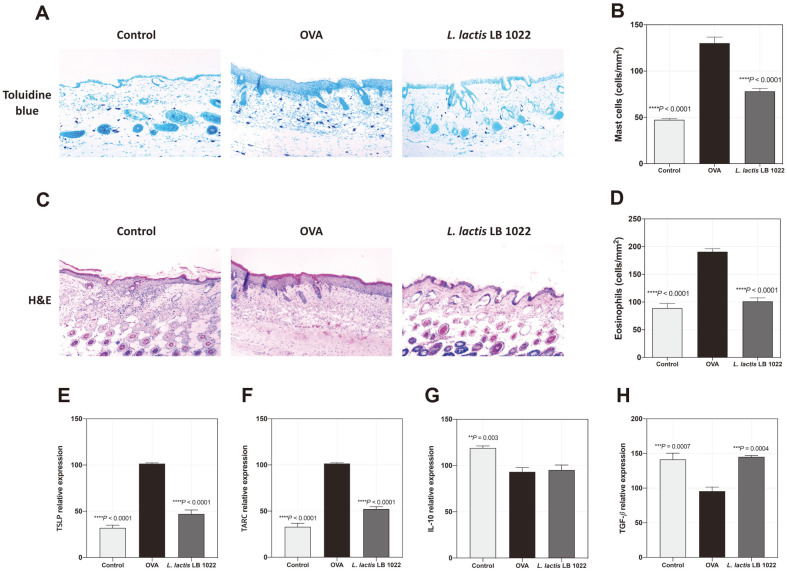
Effects of the oral administration of *L. lactis* LB 1022 on dorsal skin inflammation in OVA-induced AD mice. (**A, B**) Mast cells in the dorsal skin of mice. Mast cells were stained with Toluidine Blue. (**C, D**) Eosinophils in the dorsal skin mice of mice. Eosinophils were stained with hematoxylin and eosin. (**E**) Relative TSLP, (**F**) TARC, (**G**) IL-10 and (**H**) TGF-β mRNA expression in skin evaluated by RT-PCR. Statistical differences between the OVA and Control/*L. lactis* LB 1022 groups were assessed using one-way ANOVA followed by Turkey’s post-hoc tests (mean ± SEM).

OVA-induced mice exhibited AD-like lesions and hyperkeratosis in the skin. Oral administration of *L. lactis* LB 1022 decreases epidermal thickness (Fig. 4A). To evaluate the skin lesions in more detail, mast cells and eosinophils were stained with Toluidine Blue and H&E, respectively. The count of mast cells and eosinophils was significantly lower in the *L. lactis* LB 1022 group compared with OVA group (Figs. 4A–4D). Furthermore, the levels of TSLP and TARC were significantly lower in the *L. lactis* LB 1022 group (Figs. 4E and 4F). Interestingly, the mRNA levels of IL-10 and TGF-β were upregulated by *L. lactis* LB 1022 treatment (Figs. 4F and 4H). Thus, *L. lactis* LB 1022 suggested that TSLP and TARC are key players in the AD-like skin phenotype following treatment.

### *L. lactis* LB 1022 Treatment Modulates Pro-Inflammatory Chemokine and Cytokine Levels

**Fig. 5:**
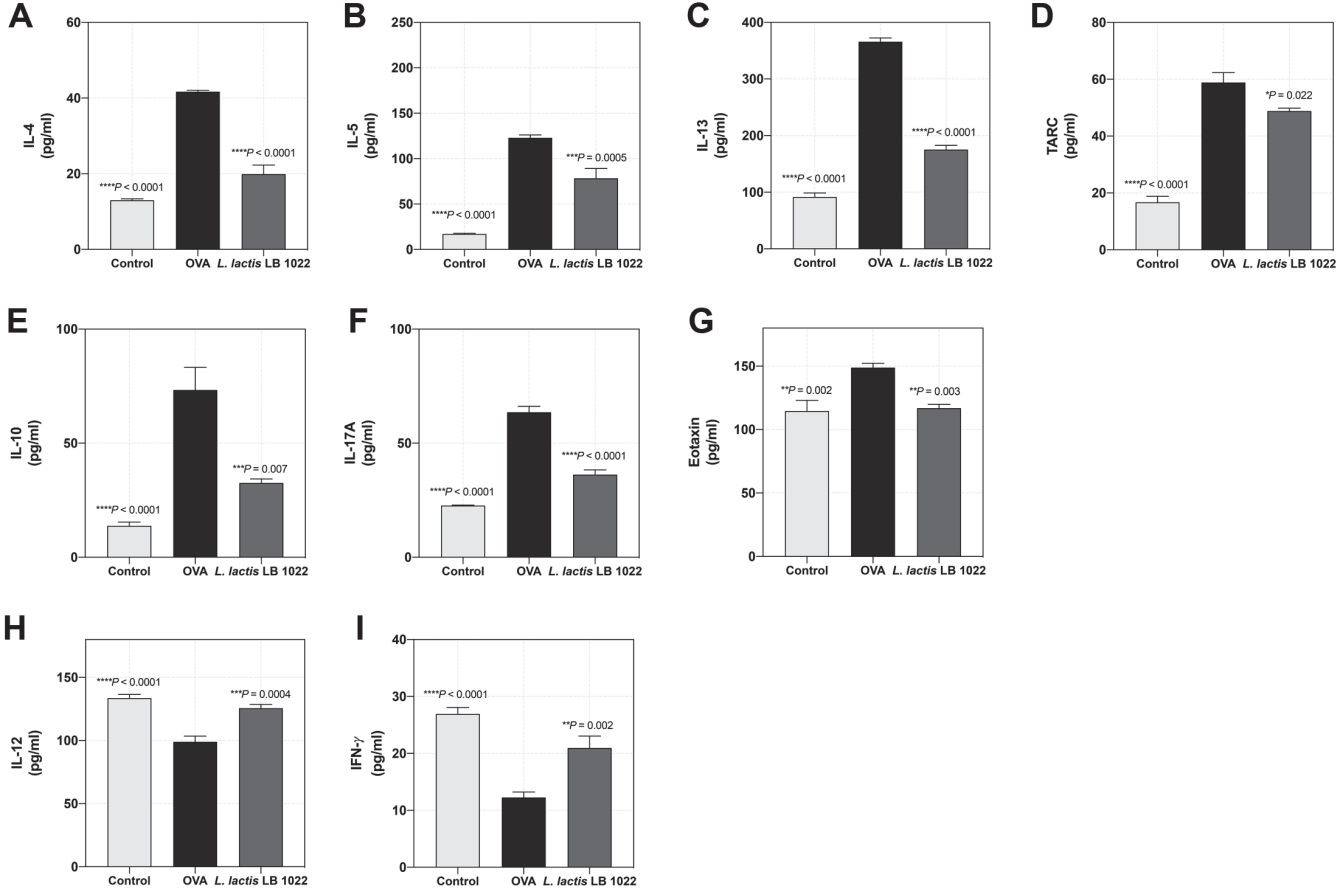
Effects of the oral administration of *L. lactis* LB 1022 on Th1, Th2, Th17, and Treg-associated cytokine production in serum. Protein levels of Th2-related cytokines (**A**) interleukin (IL)-4, (**B**) IL-5, (**C**) IL-13, and (**D**) TARC, Treg-associated cytokine (**E**) IL-10, Th17-related cytokine (**F**) IL-17A, (**G**) eotaxin, and Th1-related cytokines (**H**) IL-12 and (**I**) IFN-γ measured using an ELISA kit. Statistical differences between the OVA and Control/*L. lactis* LB 1022 groups were assessed using one-way ANOVA followed by Turkey’s post-hoc tests (mean ± SEM).

Serum levels of Th2 cytokines (IL-4, IL-5, IL-13, and TARC), a regulatory T cell (Treg) cytokine (IL-10), and a Th17 cytokine (IL-17A) were significantly suppressed in the *L. lactis* LB 1022 group compared to the control, whereas Th1 cytokines (IL-12 and IFN-γ) were present at higher levels (Figs. 5A–5F and 5H–5I). Moreover, the serum levels of eotaxin were decreased in the *L. lactis* LB 1022 group compared with OVA group (Fig. 5G).

### *L. lactis* LB 1022 Treatment Upregulated the SCFA Concentrations in OVA-Induced Mice

**Fig. 6:**
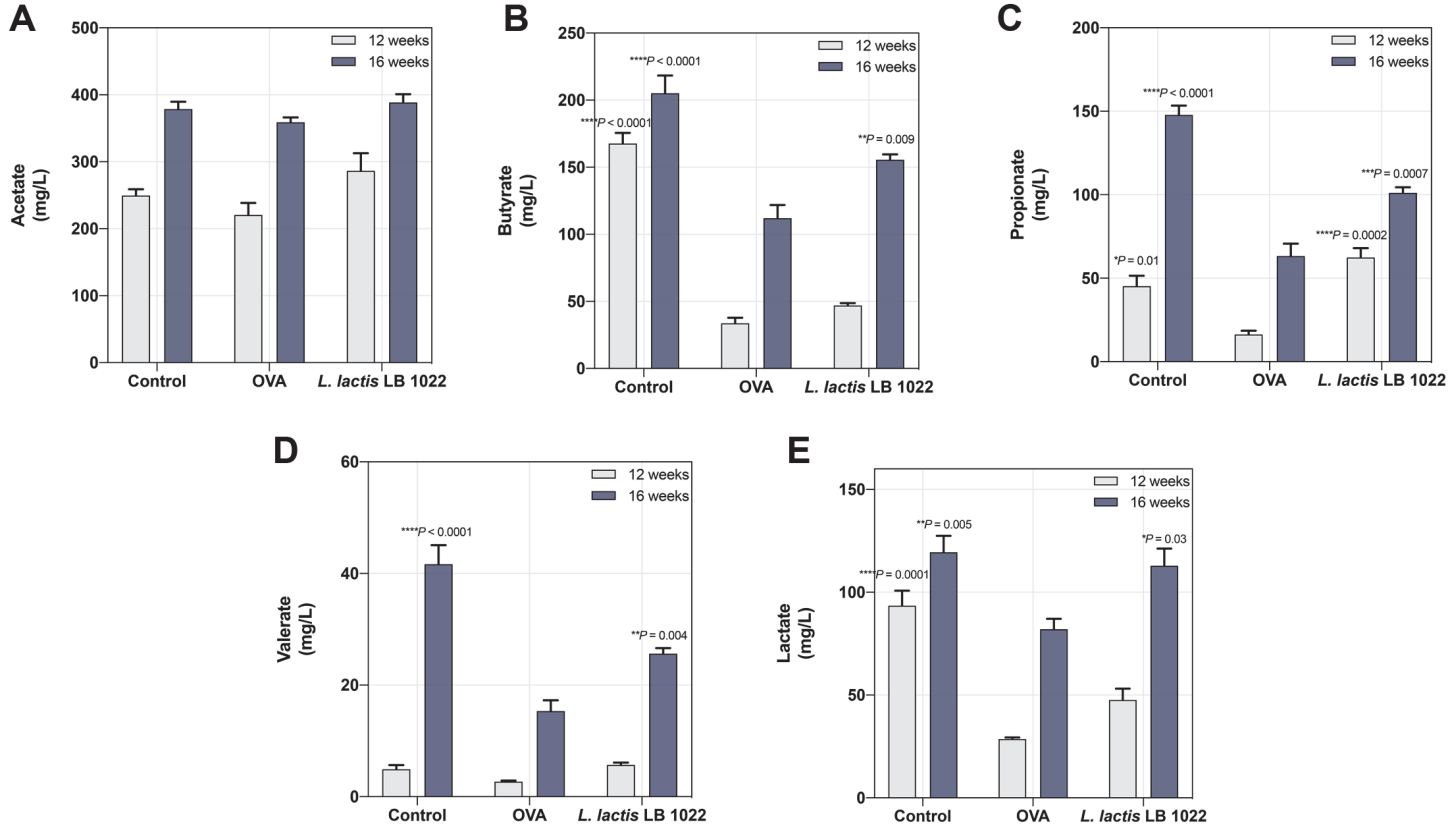
Effects of the oral administration of *L. lactis* LB 1022 on short-chain fatty acids (SCFAs) in the cecum. The concentration of (**A**) acetate, (**B**) butyrate, (**C**) propionate, (**D**) valerate, and (**E**) lactate measured using HPLC in AD mice. Statistical differences between the OVA and Control/*L. lactis* LB 1022 groups were assessed using one-way ANOVA followed by Turkey’s post-hoc tests (mean ± SEM).

The probiotic strain, *L. lactis*, is used as a fermentation substrate by gut microbiota-derived metabolites, including acetic acid, butyric acid, and propionic acid. As shown in Fig. 6, *L. lactis* LB 1022 treatment remarkably upregulated concentrations of butyrate, propionate, valerate, and lactate, but the acetate levels were not statistically different.

## Discussion

The bacterium *L. lactis* is widely used in dairy products and is extensively used to be a safety probiotic strain, with the discovery of new strains with starter culture properties further expanding its range of industrial applications [7]. In addition, various strains of *L. lactis* have recently been reported to show anti-inflammatory activity in vitro [16]. For example, oral administration of *L. lactis* ML2018 remarkably suppressed the expression of inflammatory factors in DSS-induced IBD mice [17]. However, while *L. lactis* has been widely studied in general, several studies have reported the potential effects of its strains on allergic disease. In this study, we isolated a new probiotic LB 1022 strain from natural cheese with probiotic properties, such as acid and bile tolerance and adherence. LB 1022 exhibited stronger anti-allergy and inflammatory characteristics than the control and was orally administered to an OVA-induced AD mice model to investigate whether it can alleviate AD-like lesions.

Previous studies have shown that dysbiosis of the intestinal microbiota leads to the development of atopic diseases by affecting Th2-related immune responses [18, 19]. Administration of *L. lactis* LB 1022 improved clinical AD symptoms, decreased serum IgE and suppressed the Th2 cytokines secretion, such as IL4, IL-13, and TSLP in blood, which are factors known to be elevated by AD. In our previous study, the oral administration of *Lactococcus* strains suppressed clinical phenotypes, improved gut microbial-derived metabolites, and regulated Th1/Th2 cytokines in AD mice [20, 21]. Similar to these results, *L. lactis* LB 1022 may have a protective effect against AD by reducing high IgE serum levels and Th2-related responses that arise from an imbalance in the gut microbiota.

AD is an inflammatory disease with complex pathogenesis that includes immune dysregulation. Innate immunity mechanisms, such as the regulation of Th1, Th2, Th17, and Treg, play a central role in the development of AD. In the present study, *L. lactis* LB 1022 suppressed cytokine levels associated with the Th2 (IL-4, IL-5, and IL-13), Th1 (IL-12 and IFN-γ), and Treg (IL-10) immune response, while also suppressing the production of TSLP/CCL17 and eotaxin/CCL11 compared with OVA group. Moreover, mRNA expression levels of TSLP and TARC were lower in the dorsal skin. Skin lesions in AD pathogenesis exhibit biphasic inflammation with initial Th2 and chronic Th1 phases [22]. In the initial stage of tissue inflammation, the Th2 immune response contributes to the secretion of IL-4, IL-5, and IL-13 with increased IgE levels [23]. IL-4 plays a crucial role in the pathogenesis of AD via Th2 immunity, IgE switch, and mast cell and eosinophil recruitment, which are involved in itching, dryness and erythema during allergic antigens exposure [24].

Th1 immune responses, including IFN-γ and IL-12, are higher in chronic AD symptoms, with elevated Th2 cytokine levels in acute AD lesions [23]. In addition, Tregs are closely associated with immune suppression and are essential to the control of allergic responses [25]. In the allergic response, Tregs migrate from inflammatory tissue to draining lymph nodes and regulate the inhibition of the Th2 immune response [26]. Consequently, our results suggest that *L. lactis* LB 1022 ameliorates the symptoms exhibited by OVA-induced AD mice by alleviating the imbalance in Th1/Th2 and suppressing allergic inflammation.

SCFAs, including acetate, butyrate, propionate, valerate, and lactate, are the main metabolites produced in the intestine from the microbial fermented dietary fiber and have a central role in maintaining gut homeostasis and the gut barrier [27]. Compromised epithelial barrier integrity has been reported to be associated with diseases such as AD, asthma, and autoimmunity. Furthermore, SCFAs have been identified to have anti-inflammatory abilities, including promoting Tregs expression [28]. Previously, we reported that OVA-induced gut dysbiosis is associated with AD by reducing SCFA production. [29]. Similarly, our results showed that OVA-induced AD mice had lower SCFA production, which recovered with the administration of *L. lactis* LB 1022. These results indicate that *L. lactis* LB 1022 can improve SCFA production and is related to the regulation of allergic responses induced by Treg and Th2 immunity. This study highlights the potential feature of *L. lactis* as a functional ingredient to ameliorate allergic dermatitis symptoms by promoting gut microbiota-derived metabolites.

## Figures and Tables

**Table 1:** Primers used for quantitative real-time PCR in mice.

Gene	Primers (5’-3’)
TGF-β	Forward: GAAGGCAGAGTTCAGGGTCTT
	Reverse: GGTTCCTGTCTTTGTGGTGAA
IL-10	Forward: CGGGAAGACAATAACTGCACCC
	Reverse: CGGTTAGCAGTATGTTGTCCAGC
TARC	Forward: AGAGCTGCTCGAGCCACCAATGTA
	Reverse: CACCAATCTGATGGCCTTCTTCAC
Eotaxin	Forward: GCGCTTCTATTCCTGCTGCTCACGG
	Reverse: GTGGCATCCTGGACCCACTTCTTC
TSLP	Forward: TGCAAGTACTAGTACGGATGGGGC
	Reverse: GGACTTCTTGTGCCATTTCCTGAG

## References

[1] Zhu TH, Zhu TR, Tran KA, Sivamani RK, Shi VY (2018). Epithelial barrier dysfunctions in atopic dermatitis: a skin-gut-lung model linking microbiome alteration and immune dysregulation. Br. J. Dermatol..

[2] Petersen EBM, Skov L, Thyssen JP, Jensen P (2019). Role of the gut microbiota in atopic dermatitis: A systematic review. Acta Derm. Venereol..

[3] Lee SY, Lee E, Park YM, Hong SJ (2018). Microbiome in the gut-skin axis in atopic dermatitis. Allergy Asthma Immunol. Res..

[4] Yamamoto K, Yokoyama K, Matsukawa T, Kato S, Kato S, Yamada K (2016). Efficacy of prolonged ingestion of Lactobacillus acidophilus L-92 in adult patients with atopic dermatitis. J. Dairy Sci..

[5] Reddel S, Del Chierico F, Quagliariello A, Giancristoforo S, Vernocchi P, Russo A (2019). Gut microbiota profile in children affected by atopic dermatitis and evaluation of intestinal persistence of a probiotic mixture. Sci. Rep..

[6] Anania C, Brindisi G, Martinelli I, Bonucci E, D'Orsi M, Ialongo S (2022). Probiotics function in preventing atopic dermatitis in children. Int. J. Mol. Sci..

[7] Song AA, In LLA, Lim SHE, Rahim RA (2017). A review on *Lactococcus lactis*: from food to factory. Microb. Cell Fact..

[8] Ghasemzadeh J, Shekooh Saljughi Z, Akbary P, Hasani M (2018). Effects of dietary probiotic, *Lactococcus lactis* "subspecies PTCC 1403" on the growth parameters and survival rate of grey mullet (*Mugil cephalus* L.) against *Lactococcus garvieae* bacteria. J. Anim. Environ. Sci..

[9] Yoshida A, Aoki R, Kimoto-Nira H, Kobayashi M, Kawasumi T, Mizumachi K (2011). Oral administration of live *Lactococcus lactis* C59 suppresses IgE antibody production in ovalbumin-sensitized mice via the regulation of interleukin-4 production. FEMS Immunol. Med. Microbiol..

[10] Cervantes-Garcia D, Jimenez M, Rivas-Santiago CE, Gallegos-Alcala P, Hernandez-Mercado A, Santoyo-Payan LS (2021). *Lactococcus lactis* NZ9000 prevents asthmatic airway inflammation and remodelling in rats through the improvement of intestinal barrier function and systemic TGF-beta production. Int. Arch. Allergy Immunol..

[11] Konkit M, Choi WJ, Kim W (2015). Alcohol dehydrogenase activity in *Lactococcus chungangensis*: application in cream cheese to moderate alcohol uptake. J. Dairy Sci..

[12] Frank JA, Reich CI, Sharma S, Weisbaum JS, Wilson BA, Olsen GJ (2008). Critical evaluation of two primers commonly used for amplification of bacterial 16S rRNA genes. Appl. Environ. Microbiol..

[13] Robbins KS, Greenspan P, Pegg RB (2016). Effect of pecan phenolics on the release of nitric oxide from murine RAW 264.7 macrophage cells. Food Chem..

[14] Mosmann T (1983). Rapid colorimetric assay for cellular growth and survival: application to proliferation and cytotoxicity assays. J. Immunol. Methods.

[15] Kim JH, Kim W (2022). Alleviation effects of *Rubus coreanus* Miquel root extract on skin symptoms and inflammation in chronic atopic dermatitis. Food Funct..

[16] Luerce TD, Gomes-Santos AC, Rocha CS, Moreira TG, Cruz DN, Lemos L (2014). Anti-inflammatory effects of *Lactococcus lactis* NCDO 2118 during the remission period of chemically induced colitis. Gut Pathog..

[17] Liu M, Zhang X, Hao Y, Ding J, Shen J, Xue Z (2019). Protective effects of a novel probiotic strain, *Lactococcus lactis* ML2018, in colitis: in vivo and in vitro evidence. Food Funct..

[18] Penders J, Stobberingh EE, van den Brandt PA, Thijs C (2007). The role of the intestinal microbiota in the development of atopic disorders. Allergy.

[19] Kim HJ, Lee SH, Hong SJ (2020). Antibiotics-induced dysbiosis of intestinal microbiota aggravates atopic dermatitis in mice by altered short-chain fatty acids. Allergy Asthma Immunol. Res..

[20] Choi WJ, Konkit M, Kim Y, Kim MK, Kim W (2016). Oral administration of *Lactococcus chungangensis* inhibits 2,4-dinitrochlorobenzene-induced atopic-like dermatitis in NC/Nga mice. J. Dairy Sci..

[21] Kim JH, Kim K, Kim W (2019). Cream Cheese-Derived *Lactococcus chungangensis* CAU 28 modulates the gut microbiota and alleviates atopic dermatitis in BALB/c mice. Sci. Rep..

[22] Bieber T (2022). Atopic dermatitis: an expanding therapeutic pipeline for a complex disease. Nat. Rev. Drug Discov..

[23] Yamanaka K-i, Mizutani H (2011). The role of cytokines/chemokines in the pathogenesis of atopic dermatitis. Curr. Probl. Dermatol..

[24] Brandt EB, Sivaprasad U (2011). Th2 cytokines and atopic dermatitis. J. Clin. Cell. Immunol..

[25] Smits HH, Engering A, van der Kleij D, de Jong EC, Schipper K, van Capel TM (2005). Selective probiotic bacteria induce IL-10-producing regulatory T cells in vitro by modulating dendritic cell function through dendritic cell-specific intercellular adhesion molecule 3-grabbing nonintegrin. J. Allergy Clin. Immunol..

[26] Zhang N, Schroppel B, Lal G, Jakubzick C, Mao X, Chen D (2009). Regulatory T cells sequentially migrate from inflamed tissues to draining lymph nodes to suppress the alloimmune response. Immunity.

[27] Koh A, De Vadder F, Kovatcheva-Datchary P, Backhed F (2016). From dietary fiber to host physiology: Short-chain fatty acids as key bacterial metabolites. Cell.

[28] Maslowski KM, Vieira AT, Ng A, Kranich J, Sierro F, Yu D (2009). Regulation of inflammatory responses by gut microbiota and chemoattractant receptor GPR43. Nature.

[29] Kim JH, Kim K, Kim W (2021). Gut microbiota restoration through fecal microbiota transplantation: a new atopic dermatitis therapy. Exp. Mol. Med..

